# Peer Review of “A Framework for a Statistical Characterization of Epidemic Cycles: COVID-19 Case Study”

**DOI:** 10.2196/27260

**Published:** 2021-03-18

**Authors:** Mo Salman

**Affiliations:** 1 College of Veterinary Medicine and Biomedical Sciences Colorado State University Fort Collins, CO United States

**Keywords:** COVID-19, pandemics, infection control, models, experimental, longitudinal studies, statistical modeling, epidemiology

*This is a peer review submitted for the paper “A Framework for a Statistical Characterization of Epidemic Cycles: COVID-19 Case Study.”*

## Round 1 Review

### General Comments

It seems that the aim of this submission [1] is to report a study conducted to show an approach for normalization epidemic curves from various countries using retrospective data, particularly from the city of Rio de Janeiro. The submission lacks a recognized structure to present a study with its aim and details of data sources. Furthermore, the submission includes some terms that are not appropriate for describing infectious disease in a population such as contamination and contamination cycle instead of exposure and infection rates.

### Specific Comments

1. The aim of the study should be stated in a precise statement with supportive ways to test the underlying hypothesis;
2. Details of the analytical approach should be given with its assumptions and limitations;
3. Sources of the data with overall reliability can be detailed;
4. Use the appropriate and conventional terms of infectious diseases by checking the contents of the submission with reliable epidemiologists.

## Round 2 Review

I am satisfied with the modifications to the new version. Almost all of my concerns were addressed in the new version. I will let the readers decide about the validity of the model since the authors elaborated on the approach.
